# THERAPEUTIC EFFICACY OF INTRA-ARTICULAR DELIVERY OF ENCAPSULATED HUMAN MESENCHYMAL STEM CELLS ON EARLY STAGE OSTEOARTHRITIS

**DOI:** 10.22203/eCM.v037a04

**Published:** 2019-01-29

**Authors:** J.M. McKinney, T.N. Doan, L. Wang, J.N. Deppen, D.S. Reece, K.A. Pucha, S.C. Ginn, R.D. Levit, N.J. Willett

**Affiliations:** 1 Research Service, VA Medical Center, Decatur, GA, USA; 2 Department of Orthopedics, Emory University, Atlanta, GA, USA; 3 Department of Medicine, Division of Cardiology, Emory University, Atlanta, GA, USA; 4 Wallace H. Coulter Department of Biomedical Engineering, Georgia Institute of Technology and Emory University, Atlanta, GA, USA; 5 Parker H. Petit Institute for Bioengineering and Bioscience, Georgia Institute of Technology, Atlanta, GA, USA

**Keywords:** Osteoarthritis, medial meniscal transection, human mesenchymal stem cells, cellular encapsulation, osteophytes, paracrine signaling, contrast enhanced micro-computed tomography

## Abstract

Mesenchymal stem cells (MSCs) represent a great therapeutic promise in pre-clinical models of osteoarthritis (OA), but many questions remain as to their therapeutic mechanism of action: engraftment *versus* paracrine action. Encapsulation of human MSCs (hMSCs) in sodium alginate microspheres allowed for the paracrine signaling properties of these cells to be isolated and studied independently of direct cellular engraftment. The objective of the present study was to quantitatively assess the efficacy of encapsulated hMSCs as a disease-modifying therapeutic for OA, using a medial meniscal tear (MMT) rat model. It was hypothesized that encapsulated hMSCs would have a therapeutic effect, through paracrine-mediated action, on early OA development.

Lewis rats underwent MMT surgery to induce OA. 1 d post-surgery, rats received intra-articular injections of encapsulated hMSCs or controls (*i.e*., saline, empty capsules, non-encapsulated hMSCs). Microstructural changes in the knee joint were quantified using equilibrium partitioning of a ionic contrast agent based micro-computed tomography (EPIC-μCT) at 3 weeks post-surgery, an established time point for early OA.

Encapsulated hMSCs significantly attenuated MMT-induced increases in articular cartilage swelling and surface roughness and augmented cartilaginous and mineralized osteophyte volumes.

Cellular encapsulation allowed to isolate the hMSC paracrine signaling effects and demonstrated that hMSCs could exert a chondroprotective therapeutic role on early stage OA through paracrine signaling alone. In addition to this chondroprotective role, encapsulated hMSCs augmented the compensatory increases in osteophyte formation. The latter should be taken into strong consideration as many clinical trials using MSCs for OA are currently ongoing.

## Introduction

Osteoarthritis (OA) is the most common joints’ chronic disease and remains one of the leading global causes of disability (Cisternas *et al*., 2016; Vos *et al*., 2012). Disease incidence is expected to continue rising with the increasingly obese and aging global population (Vos *et al*., 2012). OA is characterized by chronic degeneration of articular cartilage, with proteoglycan loss, chondrocyte hypertrophy, matrix fibrillation, erosion and lesion formation and eventually full-thickness loss of articular cartilage resulting in bone-on-bone contact (Bertrand *et al*., 2010; Martel-Pelletier, 2004). OA-associated phenotypic changes are not strictly limited to articular cartilage, as surrounding tissue is additionally altered, with osteophyte formation, synovial inflammation and subchondral bone remodeling (Loeser *et al.*, 2012). While physical therapy and weight loss improve functionality in patients with OA, current drugs (analgesics, viscosupplements, corticosteroid injections) only provide symptomatic relief (Messier *et al*., 2004; Yu and Hunter, 2016). There is a notable need for development of a disease-modifying therapeutic for OA. Mesenchymal stem cells (MSCs) represent a promising treatment to target the disease, relying on their regenerative capacity along with their immunomodulatory and anti-inflammatory properties.

MSC therapeutics for cartilage regeneration are widely studied, in both the pre-clinical and clinical environment. Pre-clinical studies demonstrate the efficacy of autologous, allogeneic and xenogeneic MSC intra-articular injections for cartilage regeneration (Hatsushika *et al.*, 2013; Kim *et al.*, 2014; Pigott *et al.*, 2013; Saulnier *et al.*, 2016). While varied results are reported in OA as to the efficacy of xenogeneic cell sources, this remains an understudied area (Farrell *et al.*, 2016; Saulnier *et al.*, 2016). However, recent *in vivo* data, following intra-articular injections, demonstrate that xenogeneic MSCs do not elicit an increased immune response, suggesting these cells may be immunoprivileged in the short-term (Pigott *et al.*, 2013; Saulnier *et al.*, 2016). While these pre-clinical studies show improved cartilage repair with MSC treatment, much remains unknown and MSCs have yet to be translated into an effective clinical therapy (Mamidi *et al*., 2016; Pers *et al*., 2015; Yu and Hunter, 2016; Web ref. 1). MSCs may be acting through direct engraftment, in addition to paracrine signaling, in concert with the local environment (Grigolo *et al.*, 2009; Horie *et al.*, 2012; Sato *et al*., 2012). However, MSC viability studies, following intra-articular injection, show short-term survival (~ 7 d) and small numbers of engrafted cells, indicating that the newly regenerated tissue derives mostly from the host cells (Horie *et al.*, 2012; Sato *et al*., 2012; ter Huurne *et al*., 2012). These findings suggest that the recruitment of endogenous cells may be a critical component to MSC therapies, through paracrine communication. Human MSCs (hMSCs) can secrete a wide range of paracrine factors to facilitate tissue remodeling, recruit stem and progenitor cells and modulate the immune response. In response to cartilage degeneration, hMSCs can induce tissue remodeling through secretion of factors such as interleukin (IL)-6 and stromal-cell-derived factor (SDF)-1 (Liu *et al*., 2011; Namba *et al*., 2007). hMSCs can also modulate the host immune response factors tumor necrosis factor alpha (TNF-α)-stimulated gene (TSG)-6, transforming growth factor (TGF)-β1 and adenosine (Choi *et al*., 2011; Kintscher *et al*., 2002; Kunzmann *et al.*, 2006; Shin *et al.*, 2018). Additionally, in graft-*versus*-host disease, MSC apoptosis, following delivery *in vivo* by systemic administration, further modulates the immune response; however, it is unknown if this is important in the context of local treatment of osteoarthritic joints (Galleu *et al.*, 2017). The relative contribution of hMSC paracrine factors, independent of direct cellular engraftment, is difficult to define.

Cellular encapsulation represents a promising means to isolate and study hMSC paracrine factors, independent of cellular engraftment, as the capsule provides a mechanical barrier between the encapsulated cells and the native host tissue. Sodium alginate, an inert heteropolysaccharide, is used as a vehicle to encapsulate various cell types for delivery (Orive *et al*., 2003). Sodium alginate capsules can be formed with controlled porosity, allowing encapsulated MSCs to communicate with the surrounding native tissue through paracrine mechanisms, while retaining the cells inside the microcapsules (Landázuri *et al*., 2012). Encapsulation of MSCs in semi-permeable alginate allows for diffusion of molecules into and out of the cell-containing microspheres, enabling them to sense signals from the diseased tissue and to secrete various factors into the surrounding environment. The molecular cut-off of proteins readily diffusible through the alginate capsule is ~ 80 kDa – through 24 h incubation of encapsulated fluorescent microspheres – thus allowing free transit of small cytokines, chemokines and growth factors while excluding larger proteins, such as IgG (Landázuri *et al*., 2012). In a murine hind limb ischemia model, encapsulation of xenogeneic hMSCs increases cell survival, retention and pro-angiogenic activity at the ischemic injury site and intact capsules are recoverable up to 5 months post-implantation (Landázuri *et al*., 2012). Furthermore, delivery of encapsulated xenogeneic hMSCs in a hydrogel patch post rat myocardial infarction demonstrates increased viable cell retention at the delivery site, improved cardiac function, decreased scar size and increased microvascular density (Levit *et al*., 2013). The improved retention of hMSCs using this delivery method could be explained by alginate’s partial protection from the host’s immune-mediated clearance. Encapsulation of hMSCs was used in the present study to isolate the hMSC paracrine signaling properties from direct engraftment and to study the associated effects on the onset of OA.

There are currently many ongoing clinical trials using MSCs, but these have yet to translate into an effective clinical therapy (Mamidi *et al*., 2016; Pers *et al*., 2015; Yu and Hunter, 2016; Web ref. 1). In contrast, MSCs repeatedly show great promise in pre-clinical studies (Mamidi *et al*., 2016; Pers *et al*., 2015; Yu and Hunter, 2016). This motivates more detailed investigations into the MSC mechanism of action. Particularly, the role of paracrine action *versus* direct cellular engraftment of these cells need to be explored further to better design cell-based therapies that will effectively translate to the human scale. The objective of the study was to examine the therapeutic efficacy of hMSCs with and without encapsulation in early stage post-traumatic OA. Equilibrium partitioning of a ionic contrast agent based micro-computed tomography (EPIC-μCT) was used to quantitatively analyze changes in articular cartilage, osteophyte and subchondral bone morphologies. EPIC-μCT is established and validated in reference to the gold standard in the musculoskeletal field, histopathology (Palmer *et al*., 2006; Reece *et al*., 2017; Thote *et al*., 2013; Willett *et al*., 2014; Willett *et al*., 2016; Xie *et al*., 2010). The hypothesis was that encapsulated hMSCs would have a therapeutic effect, through paracrine-mediated action, on the onset and development of early stage OA.

## Materials and Methods

### Surgical methods

Animal care and experiments were conducted in accordance with the institutional guidelines of the Atlanta Veteran Affairs Medical Center (VAMC) and experimental procedures were approved by the Atlanta VAMC Institutional Animal Care and Use Committee (IACUC) (Protocol: V004–15).

Weight-matched wild type male Lewis rats (strain code: 004; Charles River), weighing 300–350 g, were acclimatized for 1 week after they were received. A surgical instability animal model, medial meniscal tear (MMT), was used to induce OA (Bendele, 2001). Animals were anesthetized using isoflurane and injected subcutaneously with 1 mg/kg sustained-release buprenorphine (ZooPharm, Windsor, CO, USA). Skin over the medial aspect of the left femoro-tibial joint was shaved and sterilized. Blunt dissection was used to expose the medial collateral ligament (MCL), which was next transected to expose the meniscus. Then, a full-thickness cut was made through the meniscus at its narrowest point. Following transection of the meniscus, soft tissues were re-approximated and closed using 4.0 Vicryl sutures and the skin was closed using wound clips. Sham surgery was also performed in rats. For shams, the MCL was transected followed by closure of the skin without transection of the meniscus.

1 d post-surgery, MMT animals received 50 μL intra-articular injections using a 25-gauge needle. Animals were injected with i) Hanks balanced salt solution (HBSS) (*n =* 8), ii) empty sodium alginate capsules (*n =* 7), iii) 5 × 10^5^ hMSC/knee in HBSS (*n =* 8) or iv) 5 × 10^5^ encapsulated hMSC (encap hMSC)/knee (*n =* 8). The cell dose (5 × 10^5^ cells/knee) used for injection was the maximum concentration that could be encapsulated and delivered in a 50 μL volume. Sham (*n =* 8) animals were not injected post-surgery.

3 weeks post-surgery, animals were euthanized by CO_2_ inhalation. The MMT surgical model shows initial phenotypical changes associated with OA at the standard 3 weeks post-surgery time point, including cartilage damage, osteophyte formation and subchondral bone changes (Bendele, 2001). This time point was matched with animal euthanasia to assess the therapeutic effects of encapsulated hMSCs on the onset and early development of OA. Left hind limbs were collected and fixed in 10 % neutral buffered formalin.

### Cell culture and characterization

hMSCs derived from bone marrow were obtained from Emory Personalized Cell Therapy core facility at Emory University. hMSCs were cultured in complete minimum essential medium Eagle-α modification (α-MEM; 12561; Gibco) supplemented with 10 % heat-inactivated fetal bovine serum (FBS) (S11110H; Atlanta Biologicals, Lawrenceville, GA, USA), 2 mM L-glutamine (SH3003401; HyClone, Logan, UT, USA) and 100 μg/mL penicillin/streptomycin (P/S) (B21110; Atlanta Biologicals) and sub-cultured at 70 % confluency. For lentiviral preparation, HEK 293T cells (CRL-3216TM; ATCC) were cultured in complete Dulbecco’s modified Eagle’s medium (DMEM; D5546; Sigma-Aldrich). hMSC phenotype was confirmed by adipogenic, chondrogenic and osteogenic differentiation (kit protocols A1007001, A1007101, A1007201; StemPro™ Differentiation Kits, ThermoFisher Scientific). Flow cytometry was also used to characterize the hMSCs. A hMSC Verification Flow Kit (FMC020; R&D Systems) was used to confirm that MSCs expressed characteristic MSC surface markers (CD73, CD90, CD105) and lacked hematopoietic markers (CD45, CD34, CD11b, CD79A, HLA-DR).

### Encapsulation of hMSCs

1 × 10^6^ cells/mL passage 4 hMSCs were suspended in 1 % ultrapure low viscosity sodium alginate LVG (UP-LVG) (4200006; PRONOVA™ UP LVG; NovaMatrix, Sandvika, Norway). An electrostatic encapsulator (VARV1; Nisco Engineering AG, Zurich, Switzerland) with a 0.2 μm nozzle, 2.5 mL/h flow rate and 7 kV voltage was used. Capsules were gelled in 50 mM BaCl_2_. hMSC capsules were washed six times with 0.9 % saline (NaCl), re-suspended to the appropriate dose and stored at 4 °C in saline until injection. hMSC viability was confirmed with Live/Dead™ Viability/Cytotoxicity kit (L3224; Invitrogen) following encapsulation. Cell viability was quantified using ImageJ software. All rats were injected within 2 h of hMSC encapsulation.

### In vitro capsule viability study

Immediately following encapsulation and washing, encapsulated hMSCs were placed in complete α-MEM medium in 12-well plates and cultured at 37 °C, 5 % CO_2_ until different time points were reached (1, 3, 5, 7, 9, 14, 21, 28 and 35 d post plating), with medium changed every 3 d. At the specified time point, hMSC viability in capsules was determined with Live/Dead™ Viability/Cytotoxicity kit (Invitrogen).

Percentage viability was quantified by comparing the relative number of live cells stained with calcein-AM to ethidium-homodimer-stained dead cells counted through ImageJ software on serial z-stacked images, each containing 3–17 capsules, obtained by a confocal microscope, at 3.99 μm z-thickness. Image collection and quantification was completed for every 14 sections, ensuring encapsulated cells were counted only once.

### Lentiviral vector packaging

The lentiviral vector, pLenti CMV Puro LUC (w168–1) (#17477; Addgene, Cambridge, MA, USA), contains a CMV promoter upstream of the firefly luciferase gene and a murine phosphoglycerate kinase (pGK) promoter upstream of a puromycin resistance gene (Campeau *et al*., 2009). Second generation packaging plasmids were used for lentiviral packaging. pLenti CMV Puro LUC was co-transfected with a packaging plasmid, psPAX2 (#12260; Addgene) and a VSVG-envelope-expressing plasmid, pMD2.G (#12259; Addgene), in a 4 : 2 : 1 ratio into HEK 293T cells using Lipofectamine™ 3000 Transfection Reagent (L3000008; Invitrogen) and Opti-MEM™ I Reduced Serum Medium (31985062; ThermoFisher Scientific), as recommended by the manufacturer. 70 % confluent HEK 293T cells were transfected with the plasmid mixture in transfection medium (DMEM + 10 % FBS + 2 mM glutamine). Transfection medium was replaced with collection medium (DMEM + 10 % FBS + 2mM glutamine + 100 μg/mL P/S at 18 h post-transfection. Collection medium was harvested at 41, 48 and 65 h post-transfection, pooled, centrifuged at 400 ×*g* for 5 min at 4 °C and filtered through a 0.45 μm polyethersulfone membrane syringe filter (6780–2504; Whatman). The viral supernatant was concentrated using the PEG-it Virus Precipitation Solution (LV810A; System Biosciences, Palo Alto, CA, USA) and stored at − 80°C.

### hMSC lentiviral transduction

50–60 % confluency passage 3 hMSCs were transduced with 7 mL of concentrated viral supernatant in T175 flasks (353112; Corning) with 8 μg/mL polybrene (AL-118; Sigma-Aldrich) in complete DMEM containing 10 % FBS. Antibiotic selection commenced 48 h post-transduction and continued throughout culture by replacing medium with DMEM supplemented with 10 % FBS, 2 mM glutamine, 100 μg/mL P/S and 0.6 μg/mL puromycin (H9268; Sigma-Aldrich). Transduced hMSCs were passaged when reaching 60–90 % confluency. Luciferase expression was confirmed through addition of D-luciferin (L-123–250; Gold Biotechnology, St. Louis, MO, USA) to transduced hMSCs, with luminescence measurements made with a microplate luminometer (BioTek SynergyTM H1, Winooski, VT, USA). The hMSCs transduced with the lentivirus were used for bioluminescent-cell-tracking studies only. The hMSCs injected for therapeutic efficacy studies evaluated by EPIC-μCT analysis did not contain the firefly luciferase gene.

### Bioluminescence imaging (BLI)

Naïve Lewis rats were injected intra-articularly with 5 × 10^5^ cells/knee of either encapsulated or non-encapsulated luciferase-expressing hMSCs (*n =* 5 for each condition). Following cellular injections at day 0, animals received an intra-articular injection of 40 mg/mL luciferin (Promega™ Beetle Luciferin, Potassium Salts, ThermoFisher Scientific) diluted in α-MEM (12561; Gibco). Incubation times for initial and subsequent luciferin injections were optimized in a prior pilot study, in which incubation time points that yielded maximum signal were selected, using the Bruker In-Vivo Xtreme. At day 0, a 30 min incubation time was allotted before BLI was conducted using the Bruker In-Vivo Xtreme imaging system. Additional BLI readings were performed at 1, 3, 5, 7 and 9 d post hMSC injections, with subsequent luciferin injections administered 20 min (incubation time) before readings. The minimum detection limit for luciferase-expressing hMSCs, *in vitro*, using the In-Vivo Xtreme imaging system was determined to be 10,000 cells (data not shown). Bioluminescence intensity values were quantified using ImageJ software and plotted as percentage of maximum intensity. Background (naïve animals with luciferin alone) images (*n =* 4) were also collected and the averaged intensity value was subtracted from intensity values collected for all study samples.

### EPIC-μCT analysis of articular cartilage, osteophytes and subchondral cone

**Fig. 1.**
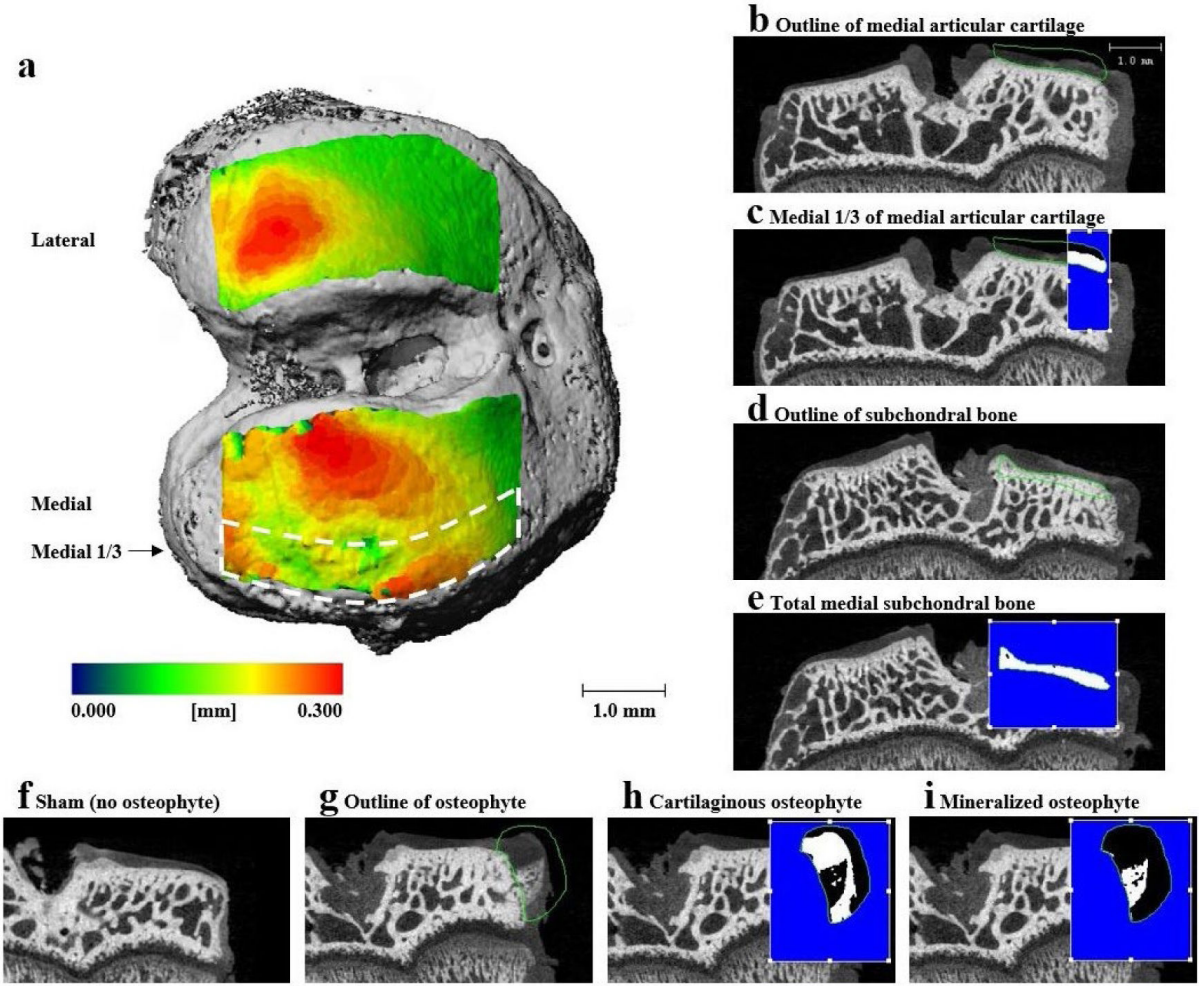
Representative EPIC-μCT images demonstrating articular cartilage, osteophyte and subchondral bone volume of interest (VOI). (a) Rat tibial articular cartilage thickness map overlays on bone indicating articular cartilage VOI. (b) Representative images of coronal sections of rat tibial joint showing outline of total medial articular cartilage, (c) medial ⅓ (indicated in white) of medial articular cartilage, outline of (d) medial tibial subchondral bone and (e) cortical subchondral bone (indicated in white) of medial tibia. Representative coronal sections of medial tibial joint illustrating (f) no osteophyte in sham joint, (g) contour of osteophyte in MMT joint, (h) cartilaginous osteophyte volume (indicated in white) in MMT joint and (i) mineralized osteophyte volume (indicated in white) in MMT joint. Scale bar in (a) is universal for all representative images of coronal sections.

EPIC-μCT was used to quantitatively evaluate articular cartilage structure and composition, osteophyte volumes and subchondral bone morphology (Fig. 1), as previously described (Palmer *et al*., 2006; Reece *et al*., 2017; Thote *et al*., 2013; Willett *et al*., 2014; Willett *et al*., 2016; Xie *et al*., 2010). Fixed tibiae were dissected, carefully exposing the articular cartilage of the medial tibial plateau and stored in phosphate-buffered saline (PBS). Before scanning, the proximal tibia was immersed for 30 min at 37 °C in 2 mL of 30 % Hexabrix® 320 contrast reagent (NDC 67684-5505-5; Guerbet, Villepinte, France) and 70 % PBS (Palmer *et al*., 2006). Tibiae were gently patted dry, placed in covered sample chambers and scanned using Scanco μCT 40 (Scanco Medical, Brüttisellen, Switzerland). Scan parameters were: 45 kVp, 177 μA, 200 ms integration time, 16 μm voxel size, ~ 27 min scan time (Palmer *et al*., 2006). Raw scan data were automatically reconstructed to two-dimensional (2D) grayscale tomograms and orthogonally transposed to yield coronal sections.

Coronal sections were evaluated in the medial ⅓ of the medial tibial plateau for articular cartilage parameters, as this region demonstrates high damage incidence in the MMT-induced OA model (Thote *et al*., 2013). Contours were constructed around medial tibial cartilaginous surfaces. Thresholding was used to separate cartilage from air and bone, on the medial ⅓, with thresholds of 110 and 435 mg hydroxyapatite per cubic centimeter (mg HA/cm^3^), respectively (Fig. 1b,c). Results yielded average articular cartilage volume, thickness and attenuation. Cartilage attenuation is a parameter inversely proportional to sulfated glycosaminoglycan (sGAG) content (Palmer *et al*., 2006). More specifically, degraded cartilage has a lower sGAG content so that a larger amount of contrast agent integrates into the cartilage matrix and, therefore, reflects a higher attenuation value (Palmer *et al*., 2006).

Osteophytes are defined as a thickening of tissue on the most medial aspect of the tibial condyle (van der Kraan and van der Berg, 2007). Osteophyte volume is evaluated using the coronal section (Reece *et al*., 2017; Willett *et al*., 2016). The osteophyte cartilage volume was measured in volumes of interest that excluded the peripheral soft tissue. For cartilaginous osteophyte volumes, threshold values of 110–435 mg HA/cm^3^ were used to segment the cartilage from air and bone. For mineralized osteophyte volume measurements, thresholds of 435–1000 mg HA/cm^3^ were used to exclude mineralized tissue volumes from surrounding cartilage, soft tissue and air (Fig. 1f–i). Total osteophyte volumes were calculated by adding the respective volumes of cartilaginous and mineralized osteophytes.

Subchondral bone was analyzed over the entire medial tibial plateau using coronal sections. Thresholds of 435–1000 mg HA/cm^3^ were used to separate the bone from the surrounding cartilage and soft tissue (Fig. 1d,e). Average subchondral bone volume, thickness, attenuation (density) and porosity were measured.

### Surface roughness analysis of articular cartilage

Serial coronal sections of the medial tibial plateau, obtained from EPIC-μCT scans, were exported as 2D grayscale TIFF images and imported into MATLAB R2017a (MathWorks, Natick, MA, USA). A custom algorithm scanned section images sequentially to create three-dimensional (3D) digital representations of respective cartilaginous surfaces (Reece *et al*., 2017). Briefly, cartilage surfaces were fitted with a 3D polynomial surface: fourth order along the ventraldorsal axis and second order along the medial-lateral axis. Surface roughness was calculated as the root mean square of differences between the articular cartilage surface and the polynomial fitted surface. Surface roughness was analyzed over the medial ⅓ of the medial tibial plateau.

### Histology

Tibiae were decalcified with Immunocal (SKU-1414–32; StatLab, McKinney, TX, USA) for 7–10 d. Dehydrated samples were processed into paraffin-embedded blocks, 5 μm-thick sectioned and stained with hematoxylin and eosin (H&E; Fisherbrand™ 517-28-2, Waltham, MA, USA) or safranin O and fast green (Saf-O; Electron Microscopy Sciences® 20800, Hatfield, PA, USA), following manufacturer protocols.

### Statistical analysis

All quantitative data are presented as mean ± standard deviation (SD). The BLI study was evaluated by repeated measures using two-way analysis of variance (ANOVA) (factors: time point and delivery method) with *post-hoc* Tukey Honest analysis. Articular cartilage and subchondral bone parameters were evaluated using one-way ANOVA with *post-hoc* Tukey Honest analysis. Osteophyte parameters were evaluated using one-way ANOVA with a nonparametric *post-hoc* Bonferroni analysis. Statistical significance was set at *p <* 0.05. All data were analyzed using GraphPad Prism software version 6.0 (GraphPad Software Inc., La Jolla, CA, USA).

## Results

### Encapsulated hMSC characterization

**Fig. 2.**
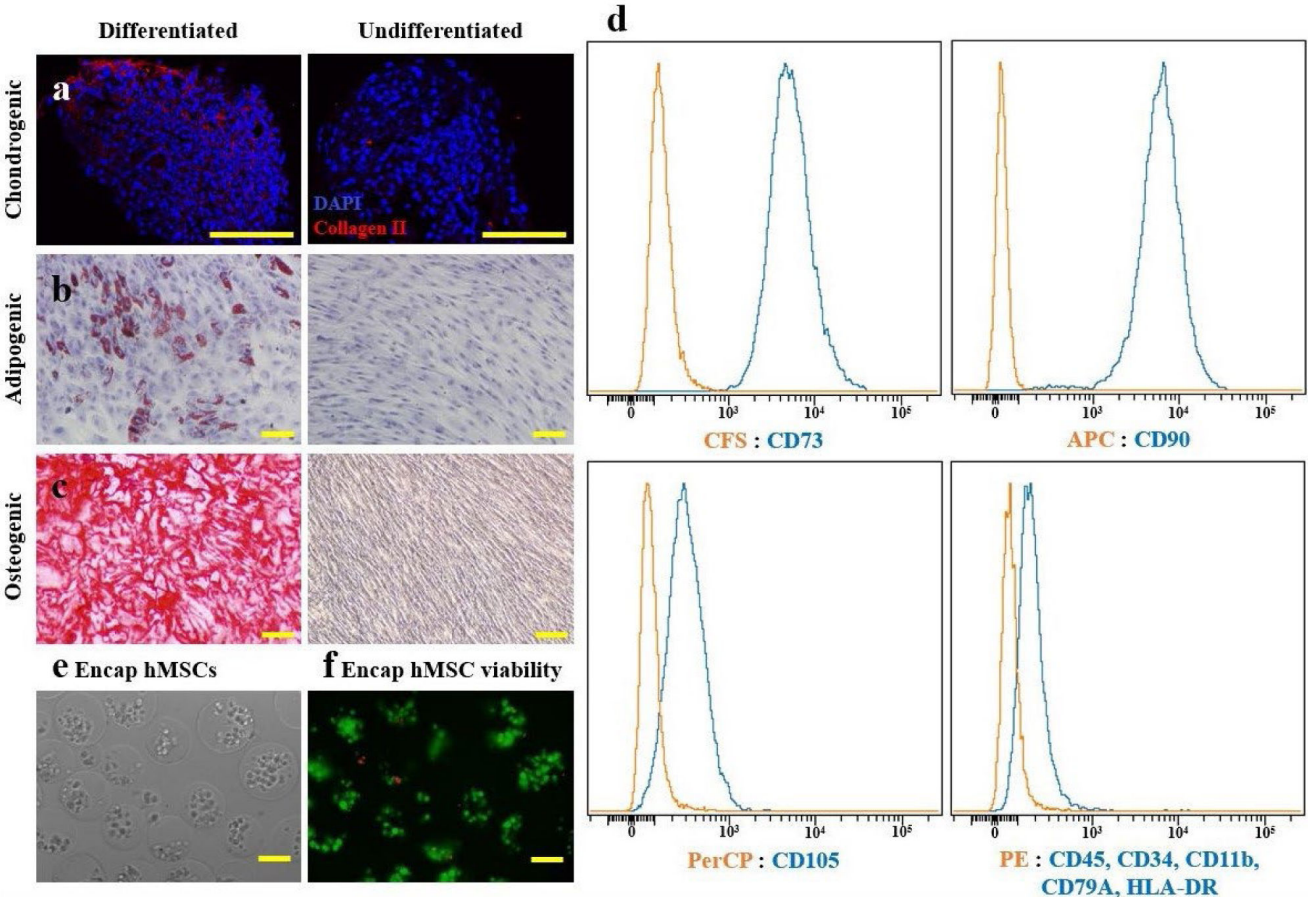
Encapsulated hMSC characterization. Multipotency of hMSCs confirmed prior to encapsulation, as cultured hMSCs were differentiated into (**a**) chondrogenic, (**b**) adipogenic and (**c**) osteogenic phenotypes as demonstrated by collagen type II, oil red O and alizarin red staining. (**d**) FACS analysis demonstrated that hMSCs expressed typical MSC surface markers: CD73, CD90 and CD105; but not hematopoietic markers: CD45, CD34, CD11b, CD79A and HLA-DR. (**e**) Light microscopic appearance of encapsulated hMSCs immediately following sodium alginate encapsulation showed capsule diameters of 170 ± 27 μm. (**f**) Fluorescent viability assay, staining live cells green and dead cells red, showed 96 ± 2.4 % of cells were viable immediately following encapsulation in sodium alginate. Scale bars = 100 μm.

To characterize the hMSCs used for therapeutic delivery, differentiation and phenotypic characterization assays were performed prior to encapsulation. hMSC differentiation was confirmed with immunofluorescence staining for type II collagen of paraffin-sectioned pellets, oil red O staining and alizarin red S staining for chondrogenesis, adipogenesis and osteogenesis, respectively (Fig. 2a–c). Additionally, hMSCs were confirmed to be positive for typical MSC markers, including CD73, CD90 and CD105, and negative for hematopoietic markers, including CD45, CD34, CD11b, CD79A and HLA-DR, by fluorescence-activated cell sorting analysis (FACS) (Fig. 2d).

hMSC were encapsulated in sodium alginate microspheres. The average diameter of encapsulated hMSC microspheres was 144 ± 16 μm (Fig. 2e). Encapsulated hMSC viability immediately following encapsulation was 96 ± 2.4 % (Fig. 2f).

### Encapsulated hMSC tracking following intraarticular injection

**Fig. 3.**
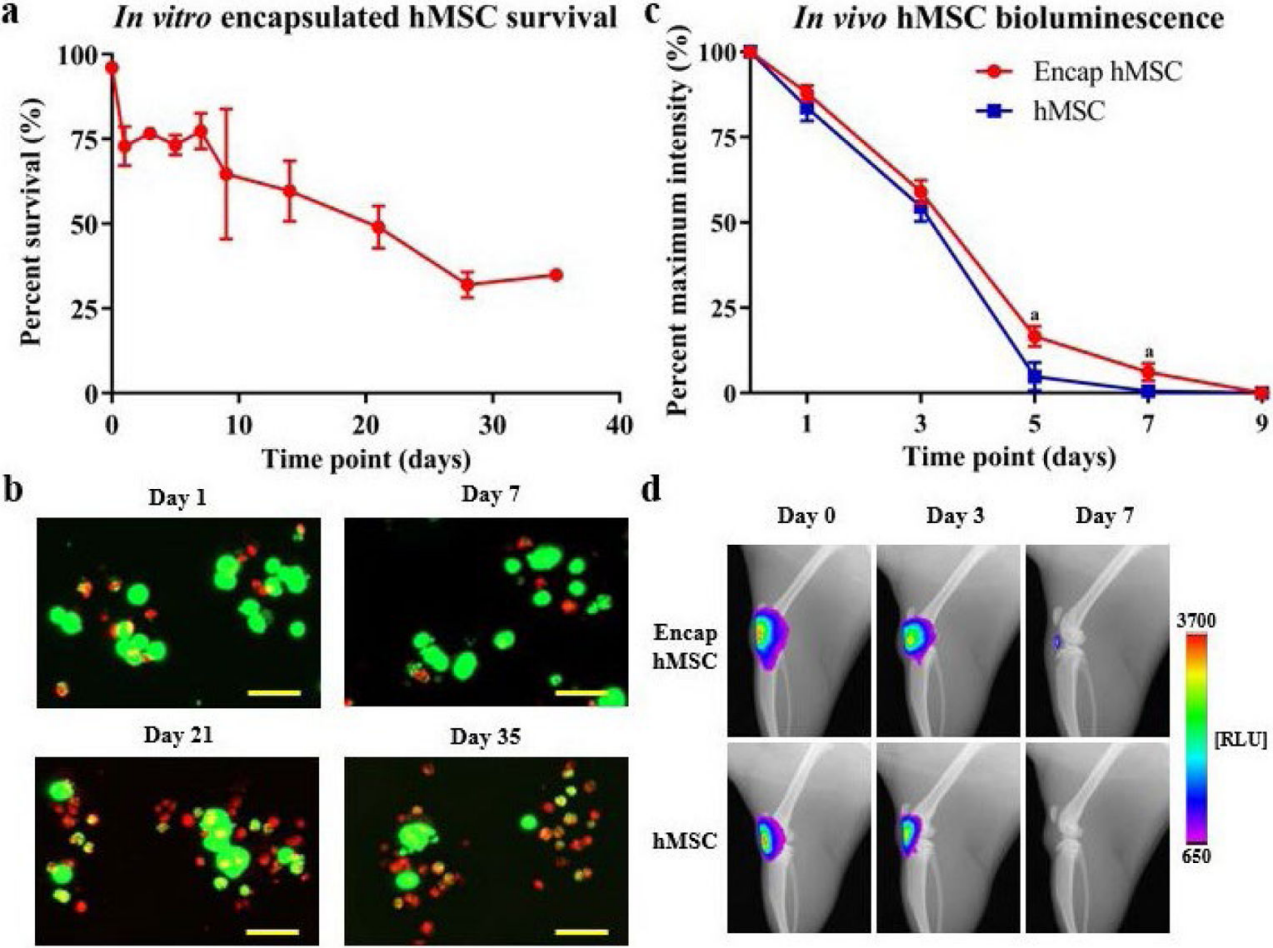
Encapsulated hMSC *in vitro* viability and *in vivo* tracking following intra-articular injection into naïve joints. (**a**) hMSCs in alginate capsules remained 70–80 % viable at early time points (days 1–7), with viability slowly declining to approximately 30 % at day 28 and 35. (**b**) Representative maximum projection images of capsules from key time points qualitatively demonstrated encapsulated hMSC viability over time *in vitro*. (**c**) *In vivo* bioluminescent imaging demonstrated an overall increase in quantified bioluminescence of encapsulated hMSCs *versus* hMSCs. Initial time points showed similar bioluminescent signals, while later time points (day 5 and 7) demonstrated differences in bioluminescent signal. Complete clearance of hMSCs (< 1 %) was observed at day 7 and complete clearance of encapsulated hMSCs was observed at day 9. (**d**) Representative images, from key study time points, of the rat knee joint qualitatively illustrate bioluminescent signal for each of the study groups. Data presented as mean ± SD. *n* = 5/group for *in vivo* study. ^a^*p <* 0.05. Scale bars = 50 μm.

Cellular encapsulation improves MSC survival in both hindlimb ischemia and myocardial infarct models (Landázuri *et al*., 2012; Levit *et al*., 2013). However, no studies show the same augmented cell survival in knee joints following intra-articular delivery of encapsulated hMSCs. Luciferase-expressing hMSCs were used to analyze the effect of encapsulation on cellular viability, retention, proliferation and metabolic state both in culture and following intraarticular injections in rat knees (Allen *et al*., 2014; Levit *et al*., 2013). *In vitro* viability studies demonstrated that encapsulated hMSCs were approximately 75 % viable for the first 7 d following encapsulation and remained approximately 30 % viable for at least 35 d under standard culture conditions (Fig. 3a). Representative maximum projection images are included displaying encapsulated cells at four key study time points (Fig. 3b).

*In vivo* bioluminescence was plotted as percentage of maximum intensity, with maximum intensity (100 %) being expressed at day 0 for all animals. Encapsulated and non-encapsulated hMSCs showed similar initial decreases in bioluminescence, as no differences in signal were detectable for the first three study time points (day 0, 1 and 3). However, at later time points (day 5 and 7), non-encapsulated hMSCs had a small but statistically significant decrease in bioluminescence when compared to encapsulated hMSCs. While complete loss of hMSC bioluminescent signal (< 1 % of original intensity) was observed at day 7, encapsulated hMSCs yielded only ~ 6 % cellular bioluminescence at this time point, with complete clearance at day 9 (Fig. 3c). Qualitative data, in the form of representative images selected from key time points for both groups, are included (Fig. 3d).

**Fig. 4.**
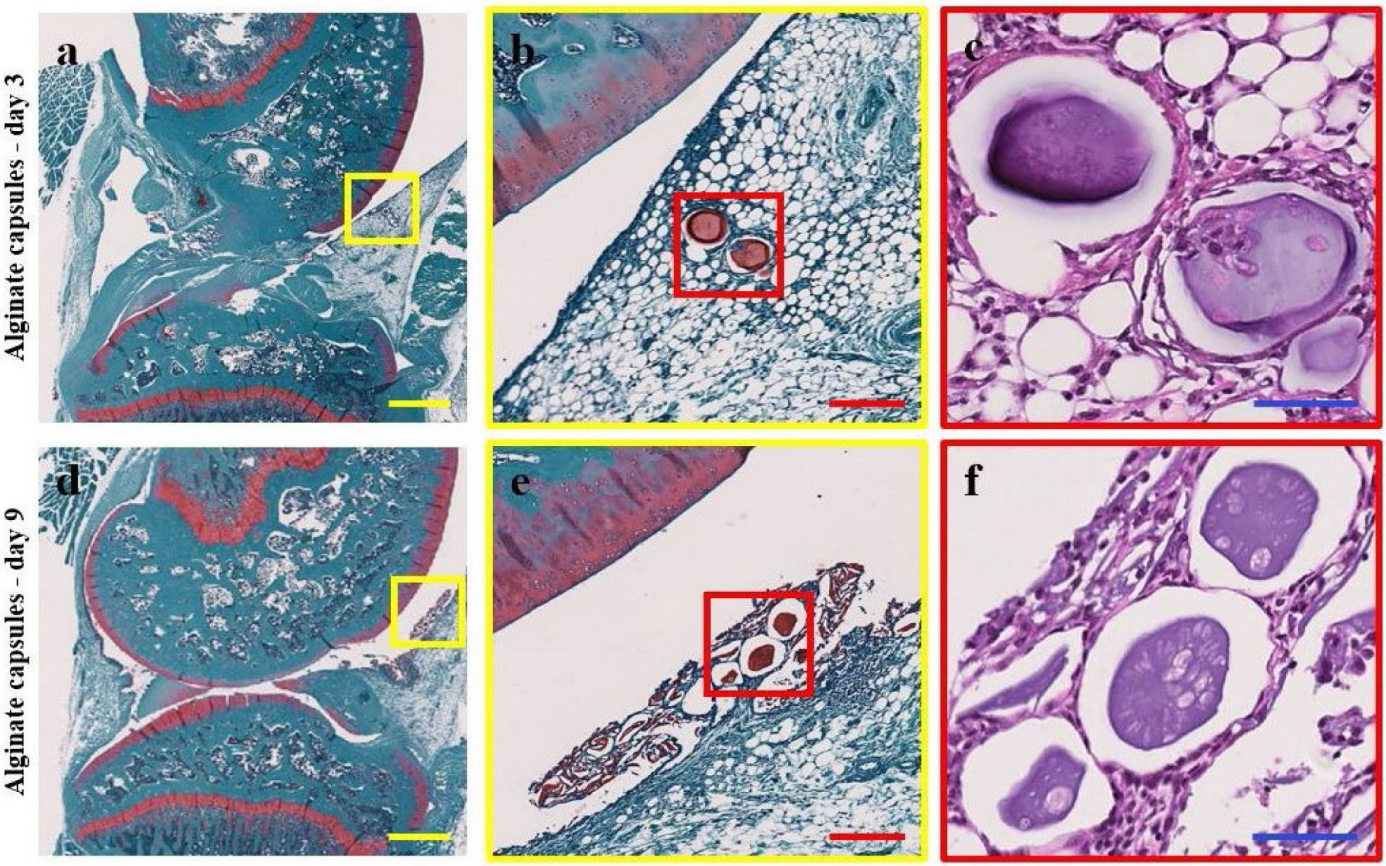
Representative histological images, single images at three separate magnifications, of full joint histology of rat hind limb knee joints at (a-c) 3 d and (d-e) 9 d post injection of encapsulated hMSCs. (**a**,**b**) At day 3, Saf-O-stained joints showed the presence of sodium alginate capsules (stained red) in the infrapatellar fat pad. (**c**) H&E staining demonstrated the presence of encapsulated cells in the sodium alginate capsules at this time point. (**d**,**e**) Saf-O staining at day 9 displayed intact sodium alginate capsules in the synovial lining of the knee joint space surrounded by remnants of broken down capsules. (f) No encapsulated cells were identifiable at day 9, as demonstrated by H&E staining, but lacunae were identifiable appearing either empty or containing cell debris. In each image, the anterior hindlimb is located on the right and the posterior hindlimb on the left. Scale bars: yellow = 1 mm, red = 200 μm, blue = 60 μm.

Full joint histology was performed on the hind limbs of animals (*n =* 2/time point) injected with encapsulated hMSCs, at 3 and 9 d post-injection, to qualitatively assess cell and capsule retention following intra-articular injection. Capsules can be readily visualized with Saf-O, a cationic stain that binds to negatively charged alginate (Schmitz *et al*. 2010). Intact alginate capsules containing hMSCs were visible within the infrapatellar fat pad of the knee at day 3 (Fig. 4a–c). Alginate capsules were also visible in the joint space at day 9, surrounded by capsule remnants (Fig. 4d,e). While no hMSCs could be identified within the capsules at day 9, lacunae empty or potentially containing cell debris were present in the capsules. Identification of encapsulated hMSCs at day 3 and the absence of hMSCs in alginate capsules at day 9 was consistent with the *in vivo* bioluminescent analysis (Fig. 3c).

### Qualitative analysis of the therapeutic efficacy of encapsulated hMSCs in OA

**Fig. 5.**
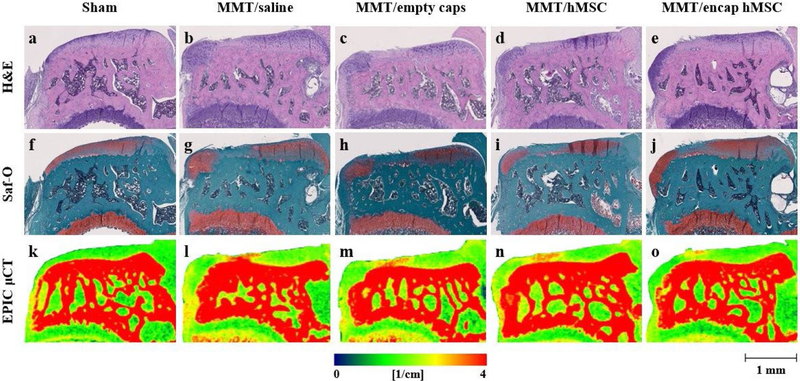
Representative histology and EPIC-μCT coronal images of medial tibial condyle following encapsulated hMSC treatment. (**a**-**e**) H&E- and (**f**-**j**) Saf-O-stained MMT joints showed no cartilage damage or osteophyte development in (**a**,**f**) sham control; (**b**-**e**,**g**-**j**) proteoglycan loss, degeneration of cartilaginous surfaces and osteophyte development in all MMT conditions. (**k**-**o**) Corresponding EPIC-μCT images showed similar disease progression as shown by histology. (**k**) No cartilage damage was observed in sham control; (**l**-**o**) increased areas of cartilage attenuation, specifically in the medial ⅓, was observed in all MMT conditions in addition to osteophyte volumes. Red indicates higher cartilage attenuation, corresponding to lower proteoglycan content. In all images, the medial tibial condyle is located on the left and the lateral tibial condyle on the right. Scale bar (bottom right corner) is universal for all histology and EPIC-μCT representative images.

Encapsulated hMSCs were injected 1 d post-surgery to assess the effects of the paracrine signaling properties of hMSCs, independent of direct cell engraftment, in delaying the onset of MMT-induced OA. Tibiae were collected 3 weeks post-surgery, the time point in the MMT model corresponding to the presentation of OA-associated phenotypical cartilage degeneration (Janusz *et al*., 2002). Histology was performed on collected tibiae to qualitatively analyze effects of encapsulated hMSC therapeutics on OA. Representative histological images of coronal tibial sections showed proteoglycan loss, degeneration of the cartilage surfaces and development of cartilaginous osteophytes in all MMT conditions (Fig. 5b–e,g–j). The sham group showed good proteoglycan staining and smooth cartilage surfaces with no osteophyte development (Fig. 5a,f). However, no further analysis was performed on H&E and Saf-O images as EPIC-μCT was used to quantitatively analyze the therapeutic efficacy of encapsulated hMSCs on OA. Representative coronal slices from both histology and EPIC-μCT showed qualitatively similar disease progression over the 3-week study time course (Fig. 5).

### EPIC-μCT quantitative analysis of articular cartilage in the MMT study

**Fig. 6.**
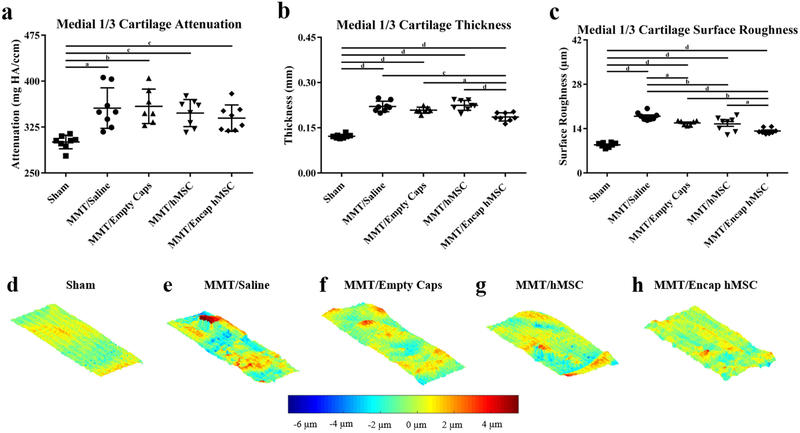
EPIC-μCT quantification of articular cartilage structure and composition in the medial ⅓ of the medial articular cartilage in MMT joints treated with encapsulated hMSCs. (**a**) Cartilage attenuation was significantly increased in all MMT conditions as compared to sham control. (**b**) Cartilage thickness was significantly increased in all MMT conditions as compared to sham control; encapsulated hMSCs attenuated increases in cartilage thickness as compared to all other MMT conditions. (**c**) Cartilage surface roughness was significantly increased in all MMT conditions as compared to sham control; saline augmented cartilage surface roughness as compared to all other MMT conditions; encapsulated hMSCs attenuated increases in cartilage surface roughness as compared to all other MMT conditions. (**d**-**h**) Representative differential surfaces illustrating surface roughness of the medial ⅓ of the articular cartilage of the medial tibial condyle. Differential maps were generated by subtracting individual 3D polynomial surfaces from the corresponding cartilage surface renderings. In these images, red indicates points that are more proximal and blue points that are further distal. (**e**-**h**) Qualitative analysis of the cartilage surfaces demonstrated increased surface roughness in all MMT conditions relative to the sham control in (**d**). (**h**) The representative surface for encap hMSCs also displayed decreased surface roughness when compared to all other MMT conditions (**e**-**g**). Data presented as mean ± SD. *n* = 7/group for MMT/empty caps; *n =* 8/group for all other conditions. ^a^*p <* 0.05; ^b^*p ≤* 0.01; ^c^*p ≤* 0.001; ^d^*p* ≤ 0.0001.

EPIC-μCT was implemented to quantitatively analyze the effects of encapsulated hMSCs on MMT-induced OA development. Changes in articular cartilage structure and composition of the medial ⅓ of the medial tibial condyle were quantified in 3D. Representative coronal cuts of contrast-enhanced cartilage EPIC-μCT attenuation maps of the whole medial tibial plateau showed qualitatively higher attenuation of cartilage for the MMT + saline (MMT/saline), MMT + empty capsules (MMT/empty caps), MMT + hMSCs (MMT/hMSC) and MMT + encapsulated hMSCs (MMT/encap hMSCs) when compared to sham control (red = high attenuation, low sGAG content; green = low attenuation, high sGAG content) (Fig. 5k–o). Quantitative analysis of EPIC-μCT images within the medial ⅓ of the medial tibial plateau showed a significant increase in attenuation, representative of a decrease in sGAG content, for all MMT conditions as compared to the sham control (Fig. 6a). However, no significant differences in attenuation were noted between any of the MMT conditions. Cartilage thickness of the medial ⅓ of the medial tibial condyle showed significant increases in thickness values for all MMT conditions in comparison to the sham control (Fig. 6b). Additionally, the encapsulated hMSCs condition yielded attenuated cartilage thickness increases in comparison to all other MMT conditions. MATLAB surface roughness analysis of the medial ⅓ of the medial tibial plateau showed an increased articular cartilage surface roughness for all MMT conditions as compared to sham animals (Fig. 6c). Furthermore, the encapsulated hMSC condition yielded attenuated surface roughness values in comparison to all other MMT conditions. Additionally, the saline group yielded augmented surface roughness in comparison to all MMT conditions. Qualitative analysis of the surface roughness was performed by subtracting individual 3D polynomial surfaces from the corresponding cartilage surface renderings, relative to the sham control (Fig. 6d–h).

### EPIC-μCT quantitative analysis of osteophytes and subchondral bone in MMT study

**Fig. 7.**
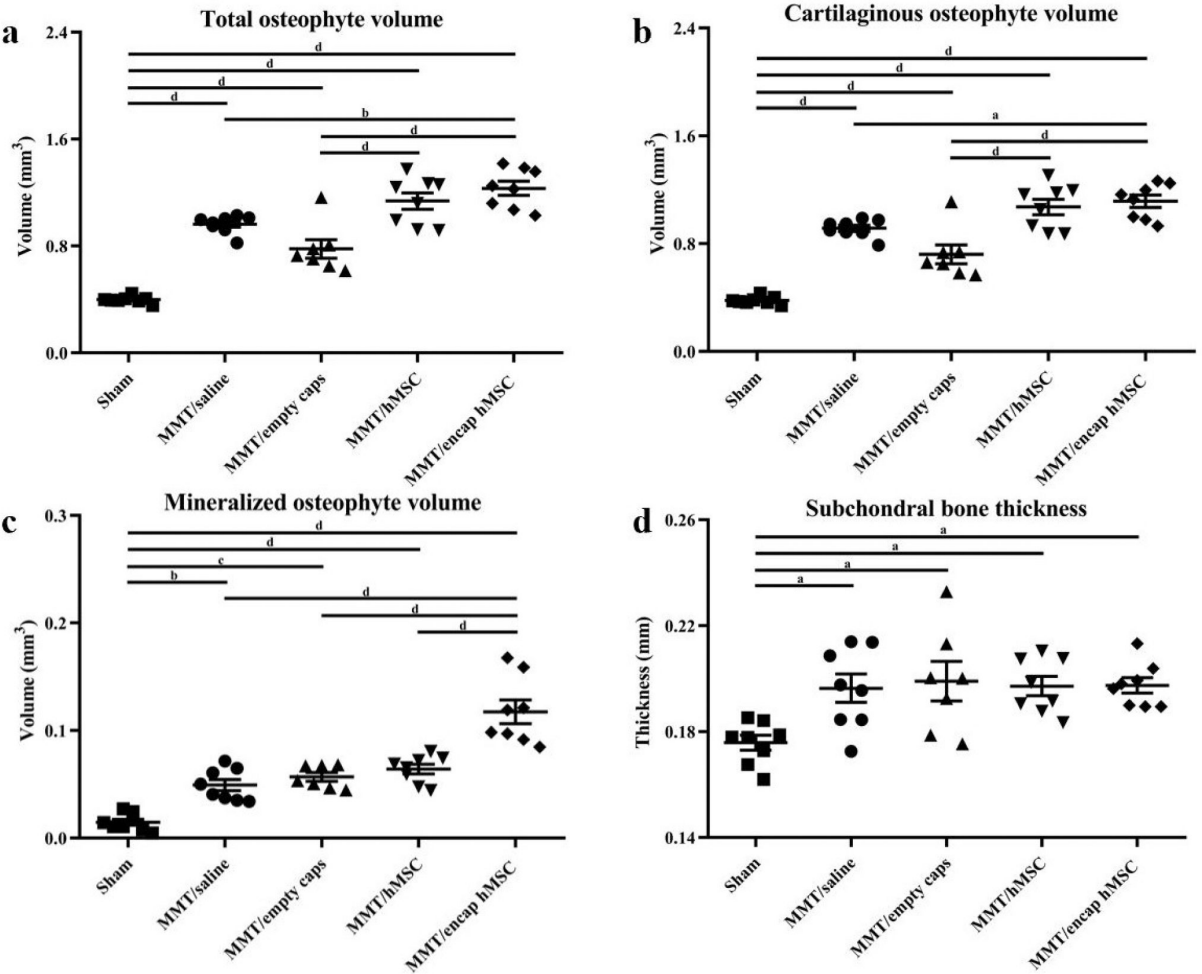
EPIC-μCT quantification of osteophyte formation and subchondral bone morphology of medial side of MMT joints treated with encapsulated hMSCs. (**a**) Total osteophyte volume was significantly increased in all MMT conditions as compared to sham control; encapsulated hMSCs augmented osteophyte volumes as compared to saline and empty capsule conditions. (**b**) Cartilaginous osteophyte volumes yielded the same respective differences among groups as the total osteophyte volume parameter. (**c**) Mineralized osteophyte volume was significantly increased in all MMT conditions as compared to sham control; encapsulated hMSCs augmented mineralized osteophyte volumes as compared to all other MMT conditions. (**d**) Subchondral bone thickness was significantly increased in all MMT conditions as compared to sham control. Data presented as mean ± SD. *n* = 7/group MMT/empty caps; *n =* 8/group for all other conditions. ^a^*p <* 0.05; ^b^*p ≤* 0.01; ^c^*p ≤* 0.001; ^d^*p* ≤ 0.0001.

Osteophytes are a thickening and partial mineralization of cartilage tissue at the marginal edge of the medial tibial plateau and are a staple of OA development (van der Kraan and van der Berg, 2007). Cartilaginous and mineralized tissue volumes on the most medial aspect of the medial tibial condyle were quantified in 3D by EPIC-μCT. Total osteophyte volume (cartilaginous + mineralized tissue volume) was significantly larger for all MMT conditions in comparison to the sham control (Fig. 7a). Furthermore, encapsulated hMSCs yielded significantly larger volumes than both the saline and empty capsule groups but not than the hMSC group. Cartilaginous osteophyte volumes were also significantly larger for all MMT conditions in comparison to the sham control (Fig. 7b). Furthermore, encapsulated hMSCs also showed increased cartilaginous osteophyte volumes in comparison to all other conditions, except hMSCs. Histological and EPIC-μCT representative images qualitatively confirmed the results of the cartilaginous osteophyte volume analysis (Fig. 5f–j,k–o). Mineralized osteophyte volumes showed an increase for all MMT conditions as compared to the sham control (Fig. 7c). Additionally, encapsulated hMSCs yielded increased mineralized osteophyte volumes in comparison to all other experimental groups. EPIC-μCT representative images qualitatively confirmed the results of the mineralized osteophyte volume analysis (Fig. 5k–o). Subchondral bone, the layer of bone just below the articular cartilage in load-bearing joints, showed an increased thickness in all MMT conditions as compared to the sham control (Fig. 7d). No significant differences were found between any of the MMT conditions for this parameter.

## Discussion

The clinical need for disease-modifying drugs for the treatment of OA remains unmet. MSCs represent a promising treatment to target OA, relying on their regenerative capacity in combination with their immunomodulatory and anti-inflammatory properties (Choi *et al*., 2011; Kintscher *et al*., 2002; Kunzmann *et al.*, 2006; Liu *et al*., 2011; Namba *et al*., 2007; Shin *et al.*, 2018). Numerous clinical trials using MSCs are ongoing, but these have yet to translate into an effective clinical therapy, despite MSCs showing great promise on the pre-clinical side (Mamidi *et al*., 2016; Pers *et al*., 2015; Yu and Hunter, 2016; Web ref. 1). This motivates more detailed investigations into the MSC mechanism of action. More specifically, the main mechanism of action of these cells following intra-articular injection into the knee space: paracrine action *versus* cellular engraftment. The objective of the study was to quantitatively assess the efficacy of encapsulated hMSCs as a disease-modifying therapeutic for early stage OA in a pre-clinical rat model.

hMSC encapsulation allows the study of the paracrine signaling properties of these cells independently of direct engraftment, as the capsule provides a mechanical barrier preventing the direct incorporation of the hMSCs into the native tissue (Landázuri *et al.*, 2012). Additionally, encapsulation improves MSC survival, which is critical for the therapeutic efficacy (Landázuri *et al.*, 2012; Leijs *et al.*, 2017; Levit *et al.*, 2013). To study the effects encapsulation would have on viability, retention, proliferation and metabolic state of hMSCs, a bioluminescence-tracking study was performed using luciferase-expressing hMSCs. While encapsulation yielded a statistically significant increase in hMSC bioluminescence, the enhancement of cell bioluminescent signal was not as pronounced as was initially hypothesized. The initial hypothesis was based on a previous work in a rat myocardial infarction model showing a sizable increase in the bioluminescent signal of the encapsulated hMSCs (Levit *et al.*, 2013). Even though Levit *et al.* (2013) show similar encapsulated hMSC bioluminescent signal detection for approximately 7 d, hMSCs alone yield an attenuated bioluminescence 1 d post-injection. However, in the present study, the bioluminescence of the non-encapsulated hMSCs (~ 7 d) was consistent with a previous report using intra-articular injections (Horie *et al.*, 2012). Van Buul *et al.* (2014) show that non-encapsulated hMSCs can survive for up to 14 d following intra-articular injections in a rat model; however, one key difference is that those results are obtained using allogeneic MSC sources. While many questions remain as to the effects of cell sourcing, particularly the utilization of an allogenic *versus* xenogeneic source, numerous reports demonstrate decreased cell variability when using xenogeneic *vs*. allogenic cells (Lin *et al.*, 2012; Niemeyer *et al.*, 2008). Furthermore, while previous studies demonstrate beneficial effects of encapsulation on hMSC viability, direct comparison of these reports to the present study was confounded by the various capsule sizes employed and the different sites of delivery employed, as each location could contain different clearance kinetics, mechanical loading and immune response components (Landázuri *et al.*, 2016; Leijs *et al.*, 2017; Levit *et al.*, 2013). In the present study, histological analysis of alginate capsule retention showed the presence of encapsulated cells at day 3 post-injection, consistent with the bioluminescent data, but cells were not identifiable in the capsules at day 9. However, at day 9, the capsules did show open spaces resembling lacunae that were either empty or contained cell debris, suggesting potential cell apoptosis. To confirm that this cell death was not due to technical issues with encapsulation, an *in vitro* study was performed demonstrating encapsulated hMSC survival for up to 35 d. While cell viability is a key component of the therapeutic efficacy of MSC treatments, recent reports suggest that hMSC apoptosis may play a key role in the immunomodulatory properties of these cells (Mamidi *et al.*, 2016; Pigott *et al.*, 2013; Galleu *et al.*, 2017). Overall, while the study demonstrated that hMSC encapsulation slightly enhanced bioluminescent signal in comparison to hMSCs alone, both groups showed no signal by day 9 post-injection. One limitation of this bioluminescent technique was that hMSC viability, retention, proliferation and metabolic state could not be studied independently. Further study will be needed to discriminate the potential individual contributions of each of these cellular properties.

To study the therapeutic effects of encapsulated hMSCs, EPIC-μCT analysis was used to quantitatively evaluate changes in articular cartilage structure and composition, osteophyte volumes and subchondral bone morphology. EPIC-μCT data showed articular cartilage lower thickness and increased surface roughness in the encapsulated hMSC group in comparison to all other MMT conditions in the early stages of OA, as the therapeutic was delivered at the time of a traumatic injury to the joint but before OA had developed. Swelling of the articular cartilage precedes fibrillation development and augmented surface roughness in the early stages of OA (Bertrand *et al*., 2010). Therefore, encapsulated hMSCs showed a chondroprotective role in early OA as they were associated with both attenuated swelling of articular cartilage and surface roughness as compared to the control groups. These findings were consistent with pre-clinical OA models showing decreased cartilage degeneration and a cartilage protective effect in MSC-treated animals (Diekman *et al*., 2013; Murphy *et al*., 2003; Toghraie *et al*., 2012). Furthermore, the chondroprotective role of MSCs in OA is reported in *ex vivo* human tissue samples with MSC treatments yielding a significant reduction in the expression of fibrotic and hypertrophic markers (Maumus *et al*., 2013). The data from the present study showed that independently of direct engraftment, hMSCs could exert a chondroprotective therapeutic effect in early OA, reinforcing the significance of the MSC paracrine signaling properties in OA treatment. One limitation of the study was the lack of secretome analysis on the hMSCs following injection; the successful recovery of cells following intra-articular delivery is technically challenging but would provide substantial information if a reliable approach would be used. This limitation motivates further study into the identification of factors being released by hMSCs when stimulated by the osteoarthritic environment. hMSC paracrine signaling properties show great therapeutic potential in protecting the joint cartilaginous surfaces in early OA but the secondary effects of these hMSC communication mechanisms must also be considered in assessing overall therapeutic efficacy.

OA-associated pathologies are not limited to phenotypic changes in the articular cartilage but can also affect the surrounding tissue, through the formation of osteophytes, synovial hyperplasia and subchondral bone sclerosis (Loeser *et al.*, 2012). Osteophytes are clinically defined as bony outgrowths – containing a fibrocartilaginous cap that forms on the margins of weight-bearing joints – and are a staple of OA development (van der Kraan and van der Berg, 2007). EPIC-μCT quantitative analysis showed an increase in mineralized osteophyte volume for the encapsulated hMSC group in comparison to all control groups. Additionally, cartilaginous osteophyte volumes were larger for encapsulated hMSCs in comparison to all groups, except the hMSC group. The effects that MSCs can have on osteophyte formation remains largely unknown. Osteophytes in general are understudied and their role and function remain poorly understood in OA. While the formation of osteophytes is hypothesized to be a compensatory mechanism in early OA, late stage osteophytes may be associated with further stages of disease progression and symptoms (Muraki *et al*., 2011; Serban *et al*., 2016; van der Berg, 1999). Even though studies as to the mechanical role of osteophytes in the knee are lacking, the function of osteophytes in the vertebral bodies of the human spines is reported (Al-Rawahi *et al*., 2011). Vertebral body osteophytes increase motion segment resistance to both bending and compression forces, suggesting that osteophytes’ formation reverses the mechanical stimuli that cause them to form, in a possible compensatory and protective role (Al-Rawahi *et al*., 2011). Clinically, in late stage knee OA, osteophyte formation strongly correlates with pain level and patient quality of life (Muraki *et al*., 2011; Serban *et al*., 2016; van der Berg, 1999); however, cartilage damage (and not osteophytes) is the strongest predictor of pain in these same late stage OA patients (Serban *et al*., 2016). Studies isolating the associated pain levels of these late stage pathologies, independent of one another, are limited (Serban *et al.*, 2016). In addition to these studies, Kirkley *et al.* (2008) demonstrate that arthroscopic removal of osteophytes brings no additional clinical benefits. The clinical implications of osteophytes are not well characterized in early OA, as osteophyte formation usually presents (or is at least identified and reported) in the latter stages of the disease, when these structures are augmented and more radiographically identifiable. While the present study did not analyze late stage OA, the results warrant further study.

Even though many questions remain as to the mechanism of osteophyte formation, MSCs are implicated as a major player in their development (van der Kraan and van der Berg, 2007). Osteophytes develop from the marginal tissue periosteum surrounding subchondral cortical bone, where endogenous MSCs are stimulated to proliferate by various mechanical and biochemical cues (van der Kraan and van der Berg, 2007). Then, cells within the developing osteophyte undergo chondrogenesis and differentiate into mature hypertrophic chondrocytes (van der Kraan and van der Berg, 2007). Following the development of this cartilaginous tissue, vascular invasion occurs and chondrocytes are replaced by osteoblasts to form bone and marrow cavities (van der Kraan and van der Berg, 2007). This developmental process is similar to the endochondral ossification in bone growth plates, however, the exact mechanism has yet to be elucidated. Fully developed osteophytes integrate with the subchondral bone and retain an outer covering of cartilage, expanding the original cartilage surface of the joint (van der Kraan and van der Berg, 2007). The implication of MSCs as a major player in osteophyte development speaks to the potential role hMSC paracrine factors may have in augmenting osteophyte formation. Numerous paracrine factors that are implicated in osteophyte development are identified in the hMSC secretome, including fibroblast growth factor (FGF)-9, TGF-β, bone morphogenic protein (BMP)-2 and insulin-like growth factor (IGF)-1 (Davidson *et al*., 2007; Okazaki *et al*., 1999; van der Kraan and van der Berg, 2007; Zhou *et al*., 2016). While many questions remain about the role of osteophytes, the results of the present study should warrant consideration and further investigation when using hMSCs in patients.

Quantitative EPIC-μCT analysis of subchondral bone thickness showed an increase in subchondral bone thickness for all MMT conditions in comparison to the sham control. This increase in thickness was evidence of increased sclerosis (abnormal hardening), which is a hallmark of OA development (Radin *et al*., 1972). However, no differences were detected between any of the MMT conditions, indicating a lack of enhancement or of a protective effect of subchondral bone sclerosis by encapsulated hMSCs. These results contrast previous studies reporting a protective effect of MSCs on subchondral bone in the form of decreased mineralization and subsequent reduction in sclerosis (thickening) (Kim *et al*., 2014; Murphy *et al*., 2003). Changes in the synovium were not assessed in the current study since significant differences in the synovium, between sham and MMT animals, do not present in this model until later time points (Kloefkorn *et al.*, 2017). Collectively, the results of the study showed that hMSC paracrine communication yielded a chondroprotective role on articular cartilage in early OA but also augmented osteophyte formations.

The hMSC group, in comparison to the encapsulated hMSC group, did not yield the same early chondroprotective effect and did not yield significantly larger mineralized osteophyte volumes than the other study groups, as measured by EPIC-μCT analysis. The results of the luciferase-tracking study suggested that the increased hMSC bioluminescence played a minor role in the therapeutic efficacy of hMSCs, as encapsulation did not enhance bioluminescent activity as markedly as was initially hypothesized. It is possible that encapsulation may also affect the paracrine signaling properties of hMSCs and further contribute to the results yielded in the EPIC-μCT analyses. However, a limitation of the study was the inability to delineate between the effects of hMSC bioluminescence (viability, retention, proliferation and metabolic state) and the paracrine signaling properties of these cells, as these could not be specifically isolated and studied independently of one another. A further limitation was the inability to successfully recover encapsulated hMSCs from the knee joint. For both the encapsulated and non-encapsulated groups, the same batch of cells were used for intra-articular injections, with both containing the same initial secretome factors. *In vitro*, encapsulation affects the MSC secretome, with certain factors being differently affected in comparison to MSC monolayer cultures (Landázuri *et al.*, 2016; Stucky *et al*., 2015). Encapsulation upregulates prostaglandin E_2_ (PGE_2_), which reduces the levels of the pro-inflammatory cytokine TNFα, implicated in the breakdown of cartilage in OA (Kunisch *et al.*, 2016; Stucky *et al*., 2015). However, the study by Stucky *et al.* (2015) is unable to delineate between the individual effects of the alginate and the 3D culture environment within the micro-capsule and the role each of these had in the study results. Furthermore, encapsulation of hMSCs upregulates numerous factors, including bFGF, phosphatidylinositol-glycan biosynthesis class F protein (PIGF) and vascular endothelial growth factor (VEGF), and downregulates the hepatocyte growth factor (HGF), among others, in comparison to non-encapsulated cells (Landázuri *et al.*, 2012). This potential for alterations in paracrine secretory activity might also account for the differences noted in the present study. Additionally, while the intercellular signaling properties of extracellular vesicles are an important mediator in the therapeutic efficacy of MSCs in OA, these structures are unable to readily diffuse through the capsules employed in the present study (Cosenza *et al.*, 2017). The two main types of extracellular vesicles, exosomes and microvesicles, which maintain sizes of 50–150 nm and 50–500 nm, respectively, exceed the 40 nm pore size of the capsules previously established (molecular diameter IgG ~ 40 nm) (Landázuri *et al.*, 2012; van Niel *et al.*, 2018). The potential effects encapsulation has on enhanced bioluminescent activity and varied paracrine secretory activity are important observations. This motivates further study of the effects encapsulation has on the secretory profiles of hMSCs and hMSCs paracrine communication and of hMSCs and encapsulation therapeutic efficacy.

With the substantial and increasing burden of OA and limited treatment options outside of temporary symptomatic relief, there is a critical need for the development of disease-modifying therapeutics. MSCs show great promise but questions remain as it relates to their mechanism of action. The present study showed that hMSCs could exert a chondroprotective therapeutic effect through paracrine signaling, independent of direct engraftment, as encapsulated hMSCs yielded an early protective role on articular cartilage in OA. Furthermore, the study showed the effects hMSCs, through their paracrine signaling properties, could have on osteophyte formation, as encapsulated hMSCs increased osteophyte volumes. These augmented tissue volumes are especially relevant in clinical applications as many clinical trials are currently ongoing but the effects of osteophyte development have not been investigated so far. Further study of the efficacy of these encapsulated hMSCs on OA progression after the disease has developed will have clinical relevance, as OA is not diagnosed clinically until patients present with augmented OA phenotypes in later stage OA.

## Conclusions

Encapsulation of bone marrow hMSCs with sodium alginate allowed for the assessment of the paracrine signaling properties of these cells, by preventing their direct engraftment into the native tissue. The paracrine mechanisms of these hMSCs showed a chondroprotective role for articular cartilage in early stage post-traumatic OA, attenuating increases in articular cartilage swelling and surface roughness. However, in addition to these early protective effects, cartilaginous and mineralized osteophyte volumes were enhanced. This early cartilage protective effect was consistent with previous reports of MSC therapeutics for OA, suggesting that direct engraftment of MSCs might not be necessary for the therapeutic benefit of an MSC injection for OA. However, the effects MSCs could have on augmenting osteophyte formations was a new observation and warrants consideration and further investigation as MSC therapies are currently being used clinically. Overall, the study demonstrated that the paracrine signaling properties of hMSCs, alone, could exert a chondroprotective therapeutic effect in early stage OA.
